# Untargeted Metabolomics Reveals a Lack Of Synergy between Nifurtimox and Eflornithine against *Trypanosoma brucei*

**DOI:** 10.1371/journal.pntd.0001618

**Published:** 2012-05-01

**Authors:** Isabel M. Vincent, Darren J. Creek, Karl Burgess, Debra J. Woods, Richard J. S. Burchmore, Michael P. Barrett

**Affiliations:** 1 The Wellcome Trust Centre for Molecular Parasitology, Institute for Infection, Immunity and Inflammation, College of Medical, Veterinary and Life Sciences, University of Glasgow, Glasgow, United Kingdom; 2 Glasgow Polyomics Facility, University of Glasgow, Glasgow, United Kingdom; 3 Pfizer Animal Health, Pfizer Inc, Kalamazoo, Michigan, United States of America; Lancaster University, United Kingdom

## Abstract

A non-targeted metabolomics-based approach is presented that enables the study of pathways in response to drug action with the aim of defining the mode of action of trypanocides. Eflornithine, a polyamine pathway inhibitor, and nifurtimox, whose mode of action involves its metabolic activation, are currently used in combination as first line treatment against stage 2, CNS-involved, human African trypanosomiasis (HAT). Drug action was assessed using an LC-MS based non-targeted metabolomics approach. Eflornithine revealed the expected changes to the polyamine pathway as well as several unexpected changes that point to pathways and metabolites not previously described in bloodstream form trypanosomes, including a lack of arginase activity and N-acetylated ornithine and putrescine. Nifurtimox was shown to be converted to a trinitrile metabolite indicative of metabolic activation, as well as inducing changes in levels of metabolites involved in carbohydrate and nucleotide metabolism. However, eflornithine and nifurtimox failed to synergise anti-trypanosomal activity *in vitro*, and the metabolomic changes associated with the combination are the sum of those found in each monotherapy with no indication of additional effects. The study reveals how untargeted metabolomics can yield rapid information on drug targets that could be adapted to any pharmacological situation.

## Author Summary

Understanding drug mode of action is of fundamental importance. Of the five drugs in use against human African trypanosomiasis (HAT), convincing evidence on a specific mode of action has been proposed only for the polyamine pathway inhibitor eflornithine. Eflornithine is currently used with nifurtimox as first line treatment of stage 2 CNS-involved HAT. Here, we present a new way of determining the mode of action of a drug by measuring how the levels of small molecules comprising the cellular metabolome are perturbed when exposed to drugs. We show that eflornithine causes the changes in polyamine metabolism previously known to underlie its mode of action. Furthermore, we show that nifurtimox, is rapidly metabolised and significantly alters metabolism. Nifurtimox and eflornithine do not show the predicted synergy with regard to trypanocidal activity and this is reflected in metabolomic analysis where perturbations to the metabolome are additive with no additional changes observed in the combination.

## Introduction

Human African trypanosomiasis (HAT) is a parasitic infection in sub-Saharan Africa transmitted by tsetse flies. Its causative agent is the flagellated protozoan *Trypanosoma brucei*, with two sub-species, *T. b. gambiense* and *T. b. rhodesiense* responsible for human disease [1], [2].

There are five drugs in use against HAT. Of these five, only eflornithine has a confirmed mode of action (MOA), namely, inhibition of ornithine decarboxylase (ODC) [3], [4] with concomitant perturbation of the polyamine pathway. In addition to the four licensed drugs, nifurtimox has been recommended by the World Health Organisation for use against late-stage disease in combination with eflornithine [5]. The MOA for nifurtimox has, however, yet to be fully elucidated. For many years it was presumed to exert its action through the generation of oxidative stress associated with reduction of the nitro group with subsequent reduction of oxygen to toxic reactive oxygen species [6], [7]. In trypanosomes polyamines serve an unusual role in combining with glutathione to create the metabolite trypanothione [8], which carries out many of the cellular roles usually attributed to glutathione in other cell types, including protection against oxidative stress. This indicated that eflornithine, which inhibits polyamine biosynthesis [9], [10] and subsequently trypanothione biosynthesis, would synergise with nifurtimox as result of a reduced ability of cells to deal with oxidative stress. However, the data that lead to the conclusion that nifurtimox causes oxidative stress is inconclusive [7] and recent evidence shows that nifurtimox is activated upon metabolism to an open chain nitrile [11] and that this nitrile is as toxic as the parent drug. In mice there was no indication that either drug enhanced uptake of the other into brain [12], indeed eflornithine diminished brain penetration of nifurtimox in short term uptake assays. Moreover, isobologram analysis indicated that the two drugs were not synergistic *in vitro* [13].

It is very rare for a new chemotherapeutic agent to be licensed without prior knowledge of its MOA. In 2009, 19 drugs were approved by the FDA's centre for drug evaluation and research in the US, only one of which had a wholly unknown MOA [14]. A knowledge of the MOA reduces the risk of unexpected toxicity and allows synergism and resistance mechanisms to be predicted. Currently, the MOA of a drug is predicted using expensive and time-consuming enzyme-based assays, followed by targeted analyses of whether cellular death is associated with changes consistent with loss of the predicted target.

Metabolomics is a relatively new technology that enables the simultaneous identification of hundreds of metabolites within a given system. In principle, if an enzyme is inhibited by a drug then the concentration of substrate should rise within a system and the concentration of product fall. We have recently introduced metabolomics approaches to investigate metabolism in trypanosomes [15]–[19]. Here we use our metabolomics platform to test the mode of action of eflornithine (an ornithine decarboxylase suicide inhibitor that has had its MOA validated) and nifurtimox (a drug for which the MOA is incompletely understood). The combination therapy was also tested to determine any synergy between the drugs at the level of metabolism. Broad changes to cellular metabolomes in response to drug have been determined before [20]–[23], and an analysis of the effect of eflornithine along with an inhibitor of s-adenosyl methionine decarboxylase were studied using a targeted multi reaction monitoring (MRM) approach in Plasmodium parasites, responsible for malaria [24] is one of few studies have focussed on individual changes that can be mapped to specific targets in the metabolic network. Here we reveal that an untargeted LC-MS based metabolomics approach identifies specific changes in the metabolome of trypanosomes that can be related directly to effects induced by these drugs.

## Methods

### Trypanosome culture

Bloodstream form trypanosomes were grown in HMI-9 (Biosera) [25] supplemented with 10% foetal calf serum (Biosera), incubated at 37°C, 5% CO_2_. Cells for metabolomics assays were grown in 500 cm^3^ Corning vented culture flasks to a maximum volume of 175 ml per flask. The Alamar blue assay developed by Raz *et al.* [26] for bloodstream form trypanosomes was used to determine activity of drugs against *T. brucei* strain 427. For isobologram analyses, alamar blue assays were conducted for one drug in the presence of three different concentrations (IC_50_, 2× IC_50_ and 0.5× IC_50_) of another drug.

### Arginase assays

A commercial kit (QuantiChrom, BioAssay Systems) was used to measure the arginase activity in cell extracts spectrophometrically (Dynex, wavelength 450 nM) by the amount of urea produced following manufacturers' specifications.

### Uptake assays

A rapid oil/stop spin protocol, previously described by Carter & Fairlamb [27], was used to determine uptake of radiolabelled ornithine (4,5-^3^H-ornithine, American Radiochemicals). Briefly, 100 µl of oil (1-Bromodo-decane, density: 1.066 g/cm^3^) (Aldrich) and 100 µl, 20 µM, 1% (v/v) radiolabelled ornithine in CBSS buffer were set up in a tube and 100 µl of cell suspension (10^8^ per mL) for varying lengths of time before stopping the reaction by centrifugation. Alternatively, radiation was used at 1% (v/v) and cold ornithine levels were varied, while keeping the incubation time constant at one minute.

The resulting cell pellet was flash frozen in liquid nitrogen and the base of the tube containing the pellet was cut into 200 µl of 2% SDS in scintillation vials and left for 30 minutes. Three ml of scintillation fluid was added to each vial and these were left overnight at room temperature.

Counts per minute were read on a 1450 microbeta liquid scintillation counter (Perkin Elmer) and normalised between samples for the cell density. Michaelis-Menten kinetic analyses were performed using Graphpad Prism 5 software.

### Metabolite extraction

Metabolite extraction methods were adapted from *Leishmania spp* extraction techniques developed previously [13], [28], [29]. Cultures were kept in log phase growth (below 1×10^6^/ml) in the presence of drug. At the time of harvest, 4×10^7^ cells were rapidly cooled to 4°C to quench metabolism by submersion of the flask in a dry ice-ethanol bath, and kept on ice for all subsequent steps. The cold cell culture was centrifuged at 1,250 RCF for 10 minutes and the supernatant completely removed. Cell lysis and protein denaturation was achieved by addition of 200 µL of 4°C chloroform∶methanol∶water (ratio 1∶3∶1) plus internal standards (theophylline, 5-fluorouridine, Cl-phenyl cAMP, N-methyl glucamine, canavanine and piperazine, all at 1 µM), followed by vigorous shaking for 1 hour at 4°C. Extract mixtures were centrifuged for two minutes at 16, 000 RCF, 4°C. The supernatant was collected, frozen and stored at −80°C under argon until further analysis.

For heavy metabolite tracking analyses, log phase cells were centrifuged 1,250 RCF for 10 minutes and resuspended in CBSS (20 mM HEPES, 120 mM NaCl, 5.4 mM KCl, 0.55 mM CaCl_2_, 0.4 mM MgSO_4_, 5.6 mM Na_2_HPO_4_, 11.1 mM glucose) or HMI-9 as outlined in the Results section. Heavy atom labelled amino acids were obtained with ^15^N incorporation from Cambridge Isotope Laboratories (L-threonine (98% enrichment, one incorporation, alpha-N, cat:NLM-742-0), L-glutamine (98% enrichment, one incorporation, alpha-N, cat: NLM-1016-0), L-arginine (98% enrichment, four incorporations, allo-N, cat: NLM-396-0), L-ornithine (98% enrichment, two incorporations, allo-N, cat: NLM-3610-0), L-lysine (95–99% enrichment, one incorporation, alpha-N, cat: NLM-143-0)) or Sigma Aldrich (L-proline (98% enrichment, one incorporation, alpha-N, cat: 608998), L-glutamate (98% enrichment, one incorporation, alpha-N, cat: 332143)). Quenching of metabolism was achieved through rapid cooling and metabolite extraction was conducted as above.

### Mass spectrometry

Samples were analysed on an Exactive Orbitrap mass spectrometer (Thermo Fisher) in both positive and negative modes (rapid switching), coupled to a U3000 RSLC HPLC (Dionex) with a ZIC-HILIC column (Sequant) as has previously been described [13]. All samples from an individual experiment were analysed in the same analytical batch and the quality of chromatography and signal reproducibility were checked by analysis of quality control samples, internal standards and total ion chromatograms. The few samples that displayed unacceptable analytical variation (retention time drift) were removed from further analysis. A standard mix containing approximately 200 metabolites (including members of the polyamine pathway) was run at the start of every analysis batch to aid metabolite identification.

### Data processing

Untargeted metabolite analysis was conducted with the freely available software packages mzMatch [30] and Ideom (http://mzmatch.sourceforge.net/ideom.html). Raw LCMS data was converted to mzXML format and peak detection was performed with XCMS [31] and saved in peakML format. MzMatch was used for peak filtering (based on reproducibility, peak shape and an intensity threshold of 3000), gap filling and annotation of related peaks. Ideom was used to remove contaminants and LCMS artefact peaks and to perform metabolite identification. Metabolite identities were confirmed by exact mass (after correction for loss or gain of a proton in negative mode or positive mode ESI respectively) and retention time for metabolites where authentic standards were available for analysis, and putative identification of all other metabolites was made on the basis of exact mass and predicted retention time of all metabolites from the KEGG, MetaCyc and Lipidmaps databases [17]. Additional manual curation was performed on all datasets to confirm the identification of metabolites that changed significantly in response to drug treatment, and to remove false-identifications based on the LCMS meta-data recorded in Ideom. In cases where identification was putative, the most likely metabolite was chosen based on available chemical and biological knowledge, however accurate isomer identification is inherently difficult with LCMS data and lists of alternative identifications and meta-data for each identified formula are accessible in the macro-enabled Ideom files (Figure S1, Figure S2, Figure S3 and Figure S4; help documentation available at mzmatch.sourceforge.net/ideom.html). Quantification is based on raw peak heights, and expressed relative to the average peak height observed in untreated cells from the same experiment.

Unidentified peaks in the LCMS data were also investigated for drug-induced changes, however, after removal of LCMS artefacts and known contaminants, the only reproducible change (<3-fold) amongst the unidentified peaks was the appearance of C_10_H_15_N_3_O_3_S (mass = 257.0834, RT = 13.5) in the nifurtimox-treated cells. This mass is in agreement with the saturated open chain nitrile metabolite of nifurtimox.

## Results

### Eflornithine and other trypanostatic compounds antagonise nifurtimox activity

**Figure 1:**
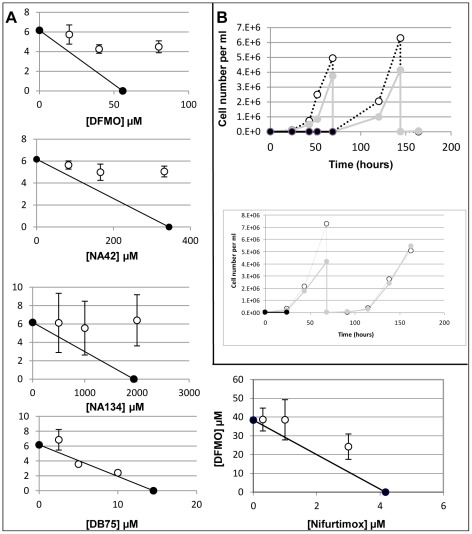
Analysis of eflornithine and nifurtimox on *T. brucei* growth. A) The effects of trypanostatic drugs in combination with nifurtimox. White points show the drugs in combination, black points show the drugs in isolation. FICs are 1.61 for eflornithine, 1.40 for NA42, 1.56 for NA134 and 1.09 for DB75 on nifurtimox action and 1.22 for nifurtimox on eflornithine action. Error bars show the standard error of the mean. N = at least 3. B) Growth curves of *T. brucei* in eflornithine (top) and nifurtimox (bottom). White points show no drug, grey shows half IC_50_ and black shows growth in toxic doses (500 µM eflornithine, 60 µM nifurtimox).

The IC_50_ of eflornithine on bloodstream form cells *in vitro* was 35 µM using a standard alamar blue assay [23]. The IC_50_ of nifurtimox was 4 µM (Table 1). The drugs were widely believed to be synergistic given the fact that eflornithine ultimately diminishes polyamine production and in turn production of trypanothione, the trypanosome's principal anti-oxidant, whilst nifurtimox had been proposed to generate oxidative stress [6], [7]. However, we showed in isobologram analyses that the action of nifurtimox and eflornithine did not synergise when nifurtimox action was assayed in the presence of several fixed concentrations of eflornithine [13] and Fig. 1A. Indeed, an antagonistic effect was seen with a fractional inhibitory concentration of 1.61.

**Table 1:** Trypanocide activities *in vitro* and in HAT treatment.

Trypanocide	Bloodstream form *in vitro* IC_50_	Human treatment
Eflornithine	35 µM [13] (*T. b. b*)3.35 µg/ml [26] (*T. b. r*)0.33 µg/ml [26] (*T. b. g*)	56 infusions over 14 days [1].
Nifurtimox	4 µM [13] (*T. b. b*)3.37 µM [44] (*T. b. b*)	3 times a day oral tablet for 10 days (only available in combination with eflornithine, 14 infusions over seven days) [52].
Pentamidine	43 nM [13] (*T. b. b*)0.2 ng/ml [26] (*T. b. r*)0.3 ng/ml [26] (*T. b. g*)	4 mg/kg daily for 7–10 days [53].
Suramin	4.6 nM [13] (*T. b. b*)19.9 ng/ml [26] (*T. b. r*)453 ng/ml [26] (*T. b. g*)	5 injections of 1 g every 3–7 days for 28 days [54].
Melarsoprol	4.3 nM [13] (*T. b. b*)1.7 ng/ml [26] (*T. b. r*)0.9 ng/ml [26] (*T. b. g*)	2.2 mg/kg daily for 10 days [1].

IC_50_s for *T. b. brucei* (*T. b. b*), *T. b. rhodesiense* (*T. b. r*) and *T. b. gambiense* (*T. b. g*) are shown.

To determine the levels at which eflornithine is cytostatic and cytotoxic, time course assays were conducted with drug at various concentrations (Fig. 1B). Eflornithine was found to be cytostatic (cells remained at the same density even at 500 µM until around 55 hours in drug, when they died). There was no overt sign of differentiation to stumpy forms, but as the 427 strain is monomorphic, and thus incapable of the morphological changes associated with differentiation in field isolates, this would not be expected. Nifurtimox, on the other hand, had lysed all trypanosomes by 8 hours in 60 µM drug. It is possible that eflornithine's antagonistic effect could relate to its cytostatic potential, if, for example, nifurtimox activity depends on cellular proliferation.

The purine analogues, NA42 and NA134 are also known to be cytostatic [32] and these compounds were tested in combination with nifurtimox and also found to be antagonistic with FICs (fractional inhibitory concentrations) of 1.40 and 1.56 for NA42 and NA134 respectively. DB75, a known potent trypanocidal agent [33], on the other hand, was shown to be additive in its activity with nifurtimox (FIC: 1.09).

### Eflornithine induced perturbation to the *T. brucei* metabolome

In order to detect molecular targets of eflornithine, a first experiment using sub-lethal levels (20 µM) of drug was used, with the cellular metabolome measured at 0, 1, 24, 48 and 72 hours following exposure to drug. Eflornithine was added to the 427 bloodstream form wild type cell line in the same growth medium in which IC_50_ values had been determined, so that cells were metabolising as normal apart from the perturbation by the drug.

The stringent filtering systems in the mzMatch and IDEOM software reduced the number of peaks in the spectra from several hundred thousand to a few hundred robust signals with putative metabolite identities (Fig. S1). Most metabolite levels were unaltered over the time points taken, indicating a high level of robustness within the trypanosome metabolome. Ornithine (mass: 132.0899, RT: 27.9 minutes), the substrate of eflornithine's known target, ornithine decarboxylase (ODC), was the most significantly modulated metabolite over the time course (7.5 fold increased at 48 hours). Putrescine (mass: 88.1001, RT: 36.91 minutes), the product of the ODC reaction was the only known metabolite in the *T. brucei* metabolite database at KEGG, to significantly decrease (by 66% at 48 hours) over time. Acetylated ornithine and putrescine were also detected, and these correlated highly with their non-acetylated counterparts. N-acetyl ornithine (mass: 174.1004, RT: 15.3 minutes) showed the most striking correlation. N-acetyl-putrescine (mass: 130.1106, RT: 15.5 minutes) was seen in early samples, but levels rapidly fell below the level of detection (1,000) from an average intensity of 41,000 (peak height) before drug addition, correlating with the decrease in putrescine.

**Figure 2:**
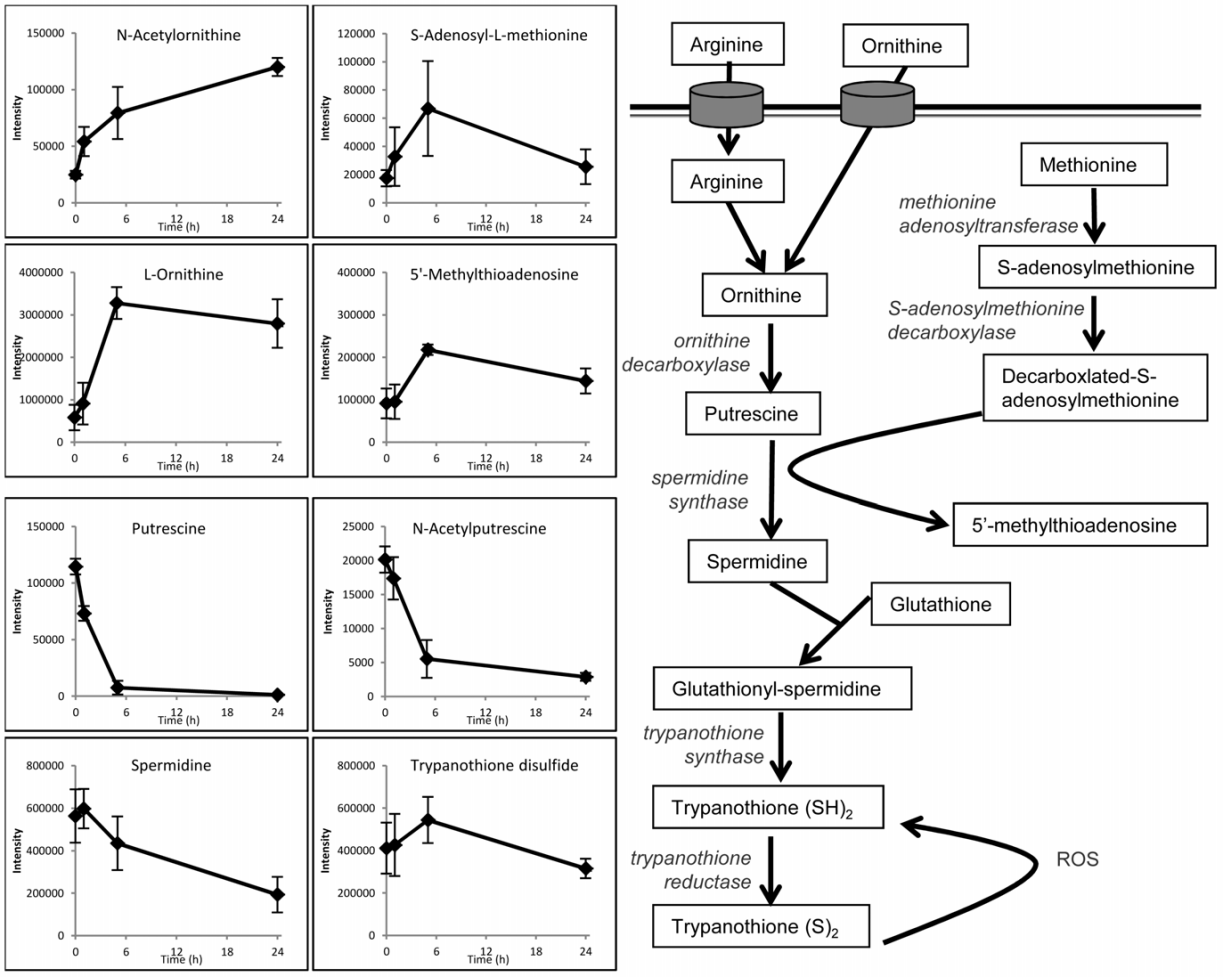
Polyamine pathway and metabolite changes after addition of toxic (500 µM) dose of eflornithine. X-axes indicate the time in hours since eflornithine addition. Y-axes indicate the raw abundance of each metabolite signal. Results show mean and standard deviation of 3 replicates.

Cells were also treated with 500 µM eflornithine, a lethal dose of the drug. At this dose bloodstream form trypanosomes exhibit division arrest over 48 hours in drug before dying between 48 and 55 hours (Fig. 1B). This was reflected by many more changes to the metabolome (Figure S2). Changes to polyamine pathway metabolites were again consistent with inhibition of ODC, with significant increases in ornithine and N-acetyl ornithine, and decreases in putrescine and N-acetyl putrescine, observed within 5 hours and maintained for the duration of treatment. Spermidine was significantly decreased by 24 hours, confirming the downstream effect of ODC inhibition on polyamine levels (Fig. 2). Additional metabolites that significantly increased within 24 hours were putatively identified as N-acetyl spermidine, N-acetyl lysine and N5-(L-1-Carboxyethyl)-L-ornithine (a known bacterial metabolite formed from ornithine and pyruvate, although we are not in a position to rule out its generation as a non-enzymatic liaison between these chemicals during sample preparation). These metabolites, along with N-acetyl ornithine, demonstrate metabolic derivitisation of ornithine and other polyamine metabolites, which may be an upregulated process in response to the elevated ornithine levels.

Aside from the polyamines, most major decreases in metabolite levels over 24 hours were observed among the phospholipids. Polyamines have previously been shown to be key mediators of membrane stability [34]–[36], and the lipid degradation observed here is consistent with cell membranes being compromised by polyamine depletion. Furthermore, the majority of metabolites in the cell decrease at the 48 hour time point, indicating a possibility that the cell membrane has been compromised and metabolites may be leaking from the cell during incubation and/or sample preparation. The processing of the cells involves cooling them to 0°C in a dry ice–ethanol bath and two centrifugation steps. These weakened cells are therefore potentially more leaky than cells that have not been compromised by prolonged exposure to eflornithine.

Several methionine-related metabolites (cystathionine, S-adenosyl methionine, methylthioadenosine and methyl-methionine) increased over the first five hours in drug, which was not reported in previous studies. S-adenosyl methionine is the aminopropyl donor involved in spermidine synthesis, and it is possible that this pathway has been upregulated in response to the declining polyamine levels. Methionine levels do not increase over this time course, however, this may be due to the high concentration of methionine in the growth medium (200 µM) and robust transport [37] masking any changes within the cells.

Despite the significant decrease observed for spermidine, levels of trypanothione disulphide were not affected during the first 24 hours of treatment. A significant decrease was observed at 48 hours. The analytical platform used here is not capable of reporting the oxidation state of trypanothione or other thiols.

The other significant changes observed during the first 24 hours of eflornithine treatment were not expected. Sedoheptulose (mass: 210.0738, RT: 14.9 minutes) and sedoheptulose phosphate (mass: 290.0400, RT: 25.4 minutes) were increased, as well as a metabolite with the chemical formula C_7_H_12_O_5_ (mass: 176.0683, RT: 7.52 minutes), putatively identified as propylmalate, but possibly diacetylglycerol or another isomer.

### Sources of ornithine in *T. brucei*

**Figure 3:**
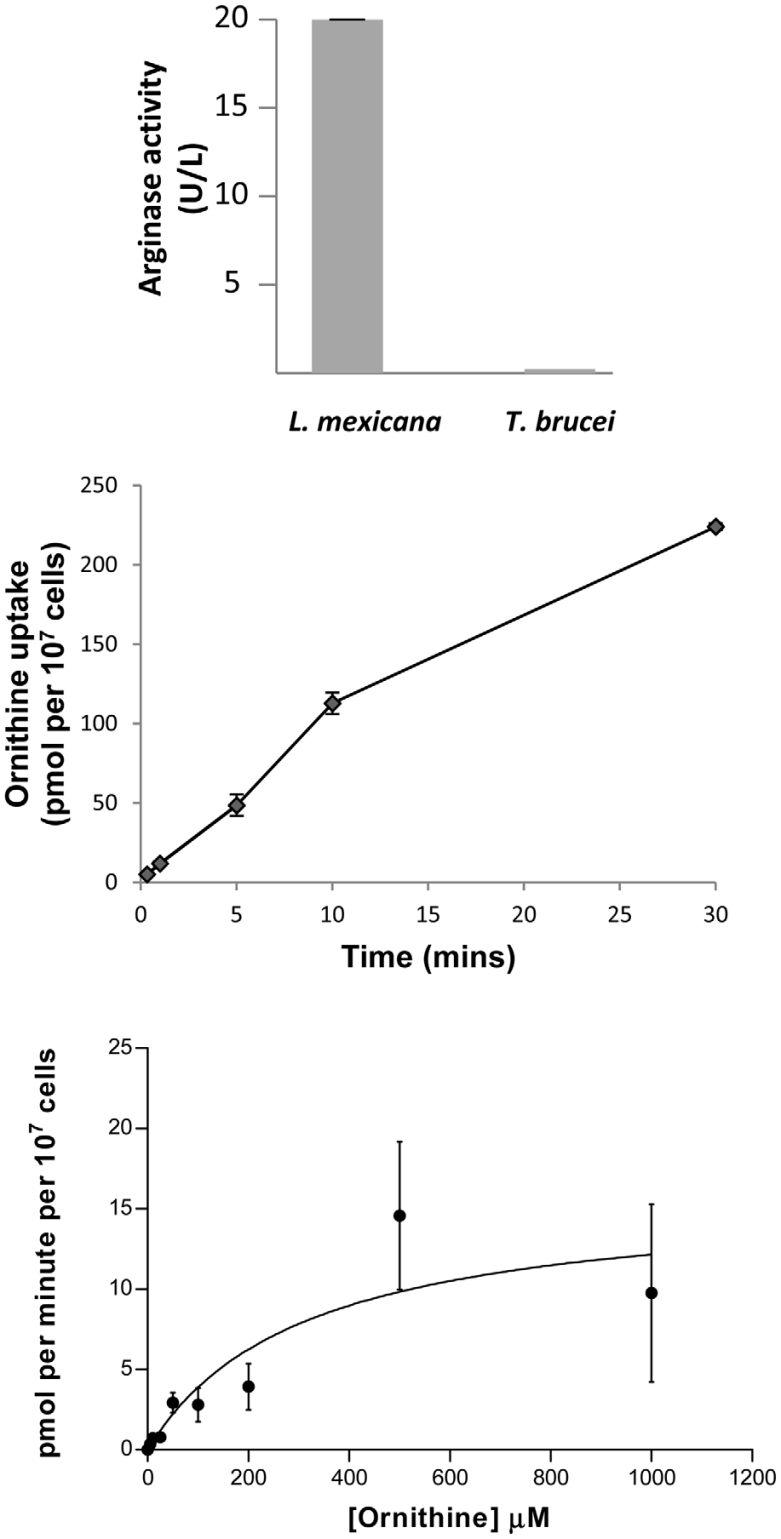
Ornithine uptake may be sufficient for polyamine synthesis. A) Arginase activity in *L. mexicana* and *T. b. brucei* cell extracts. One unit is equivalent to 1 µmole of arginine converted to ornithine and urea per minute at pH 9.5 and 37°C. N = at least 3. Results show mean ± S.E.M. B) Ornithine uptake at 20 µM over time in *T. b. brucei*. N = 4. C) Kinetics of the ornithine transporter in bloodstream form *T. b. brucei*. Km: 310 µM, V_max_: 15.9 pmol/min/10^7^ cells. Results show mean ± S.E.M, N = 4.

Our metabolomics analysis above reveals ODC to be the primary target of eflornithine, as was already clear based on previous work and the design of the compound as a specific inhibitor of the enzyme. Surprisingly, however, we could find no previous work that has focused on the cellular source of ornithine in *T. brucei*. In many eukaryotes, ornithine is produced from arginine via the enzyme arginase. In Leishmania parasites, which belong to the same taxonomic group as *T. brucei*, for example, an arginase enzyme has been characterised in some detail [38]–[41]. *T. brucei*, however, lacks a gene that is syntenic with the known Leishmania arginase. A second gene related to arginase is present in Leishmania and an orthologue is present in *T. brucei* (Tb927.8.2020). This latter predicted enzyme, however, lacks key arginase residues and is currently annotated as a putative agmatinase (although this also seems unlikely given the lack of conservation of key active site residues). We measured arginase activity in *Leishmania mexicana* extracts and compared this to *T. brucei* extracts where we show that the trypanosome contains little or no classical arginase activity when compared to Leishmania (Fig. 3A). The absence of a classical arginase raises questions about other potential sources of ornithine in *T. brucei*. Our experiments did reveal the presence of N-acetyl ornithine in *T. brucei*, the abundance of which was closely correlated to ornithine. Differences in the retention times between ornithine (RT = 27.9 minutes) and acetylornithine (RT = 15.3 minutes) confirm that the two metabolites are not mass spectrometry artefacts. In a variety of bacteria ornithine is produced from glutamate in a pathway that involves N-acetyl ornithine as an intermediate [42], [43].

We used heavy-nitrogen labelled metabolites to trace whether a similar pathway exists in *T. brucei*. However, cells incubated with isotopically-labelled extracellular ^15^N-glutamate failed to accumulate this amino acid to a detectable level. We therefore provided ^15^N labelled glutamine, which was converted to glutamate (albeit at a relatively low level of 5% of the non-labelled metabolite) after two hours and ^15^N-proline which was converted to glutamate at levels of 3.1% of the unlabelled glutamate generated within these cells. However, the heavy atom labelled glutamate was not further converted to ornithine, N-acetyl ornithine or N-acetyl glutamate semialdehyde (another metabolite of the glutamate to ornithine pathway). Furthermore, no orthologues, other than N-acetyl ornithine deacetylase (Tb927.8.1910), encoding enzymes of the bacterial pathway could be identified in the trypanosome genome indicating that this pathway is not operative in trypanosomes.

Although ornithine is not a component of HMI-9 medium, metabolomics analysis of our medium indicated that the commercial supply we used did contain ornithine and we were able to measure its concentration at 77 µM, using a calibration curve with isotopically labelled ornithine. We therefore measured the ability of ^3^H-ornithine to enter trypanosomes. This indicated a possible external source of ornithine and we tested the ability of this nutrient to enter trypanosomes. At 10 µM, ornithine was shown to enter bloodstream form *T. brucei* at a rate of approximately 10 pmol/10^7^ cells/min (Fig. 3B). Kinetic analysis of ornithine transport indicated a K_m_ of 310 µM and V_max_ of 15.9 pmol/10^7^ cells/min (Fig. 3C). Given that ornithine is present in serum and cerebrospinal fluid (at concentrations of 54–100 µM in plasma and 5 µM in CSF (according to the human metabolome database, http://www.hmdb.ca/)), this would indicate that *T. brucei* is capable of fulfilling its ornithine requirements directly by transport from the bodily fluids in which it resides. When we used ^15^N-ornithine externally to trace its metabolism we showed that N-acetyl ornithine, spermidine and trypanothione disulphide when added to cells growing in HMI-9. ^15^N-labelled arginine was converted to ornithine when administered in CBSS (Carter's balanced saline solution), but not when administered in HMI-9 growth medium. This suggested that when exogenous ornithine is present, uptake of ornithine is sufficient for polyamine synthesis, but when absent, synthesis from arginine is possible. This was confirmed by the addition of exogenous ornithine in addition to heavy arginine in CBSS, where synthesis of heavy ornithine from arginine was no longer detected (heavy ornithine being present at 40% of unlabelled ornithine levels when exogenous ornithine was not added under the same conditions). The enzymatic route by which arginine is converted to ornithine in the absence of canonical arginase is not known.

### Nifurtimox induced changes to the trypanosome's metabolome

**Figure 4:**
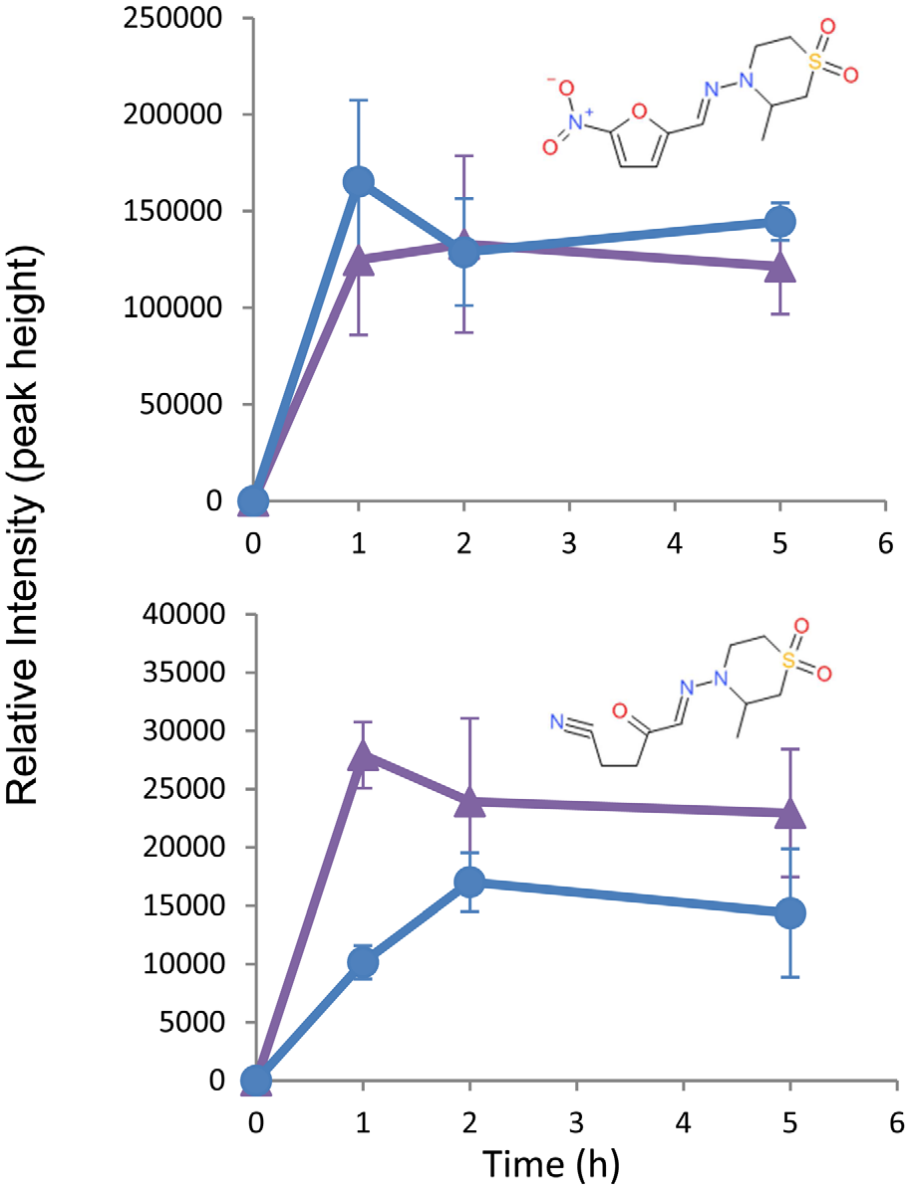
Nifurtimox metabolism to saturated open chain nitrile. Purple triangles = NECT, Blue circles = Nifurtimox. Nifurtimox (A) (Mass: 287.0576, RT: 5.4 minutes) is reduced, through a number of steps to a saturated open chain nitrile (B) (Mass: 257.0834, RT: 13.5 minutes). Neither metabolite is detected at the 0 time point (where no drug is added). N = 3. Error bars show standard deviations.

At the sub-lethal dose of 1.5 µM nifurtimox, no significant changes to the metabolome were recorded (data not shown). However, at a lethal dose of 60 µM changes to the metabolome at 0, 1, 2 and 5 hours following exposure to drug, were seen (Figure S3). Nifurtimox (mass: 287.0577, RT: 5.25 minutes) was observed in all treated samples, in addition to a mass (mass: 257.0834, RT: 13.5 minutes) consistent with the saturated open chain nitrile metabolite [11] (Fig. 4A) which was recently shown to be the end product of the multi-step 2-electron reduction of nifurtimox by type-1 nitroreductase. Previous work in a cell-free system showed the saturated nitrile only after 24 hours of drug exposure to the nitroreductase [11]. Our metabolomics platform allows identification of this metabolite within the cell, and shows the process to be rapid with significant levels detectable at the first, 1 hour time point. The implicit intermediates from this reductive activation cascade, including the unsaturated open chain nitrile proposed to mediate trypanocidal activity, were not observed, indicating either that the reduction is rapid and intermediates in the pathway do not persist at detectable concentrations, or that the reactive intermediates indeed react rapidly with intracellular macromolecules. An exhaustive search of all known metabolites in our database revealed no detectable masses that correspond to a hypothetical adduct between the unsaturated open chain nitrile and any known metabolite. Our metabolomics platform, by definition, was unable to detect the potential formation of adducts between nifurtimox metabolites and macromolecules (proteins or nucleic acids).

A number of cellular metabolites were shown to change in abundance over the nifurtimox exposure time course (Table 2), although 95% of putatively identified metabolites were stable. There was an increase in concentrations of nucleotides and nucleobases (adenine, deoxyadenosine, AMP, GMP, uracil and UMP) during the time course, which may result from degradation of RNA and DNA consistent with the hypothesis [11] that the nifurtimox active metabolite (the open chain nitrile) binds to macromolecules including nucleic acids, by the ability of the unsaturated nitrile intermediate to act as a Michael acceptor [11].

**Table 2:** Changes in metabolite abundance (relative intensity) induced by 5 hours of eflornithine, nifurtimox and NECT treatment expressed relative to untreated levels.

	Ratio 5:0 hours		
Name	Eflornithine	Nifurtimox	NEC
Ornithine	**5.62**	1.11	**2.97**
Acetylornithine	3.2	0.93	1.21
Cystathionine	**2.99**	1.09	1.13
Methylthioadenosine	**2.38**	1.11	2.06
S-adenosyl methionine	3.83	1.03	1.72
Putrescine	**0.07**	0.95	**0.16**
Acetylputrescine	0.37	0.94	1.52
Trypanothione disulphide	1.32	**0.59**	0.32
Arginine phosphate	0.83	0.47	0.52
Uracil	0.98	**2.62**	**1.93**
UMP	0.74	2.18	1.68
Adenine	1.23	5.01	6.06
AMP	0.97	**3.61**	**3.00**
GMP	1.33	**1.52**	1.25
Hexose phosphates (average of isomeric peaks)	1.31	**0.56**	0.72
3-Phosphoglycerate	1.29	0.63	**0.44**
Glyceraldehyde 3-phosphate	1.26	0.67	0.67
Deoxyribose	0.82	**0.41**	**0.18**
Peptides (average)	1.27	0.97	1.2
Lipids (average)	1.07	1.1	1.0

Bold numbers indicate a significant change according to the student's T-test (α = 0.05).

Glycolysis appeared to be downregulated, with significant decreases in hexose 6-phosphates, and similar trends for glyceraldehyde 3-phosphate and 3-phosphoglycerate. The metabolite that decreased most following nifurtimox treatment was deoxyribose, which may indicate reduced synthesis from the glycolytic intermediates, or could be related to nucleic acid homeostasis. Lipid metabolism was largely unaffected with the exception of decreased levels of monounsaturated ether-linked lysophosphatidylcholines (14∶1, 15∶1 and 16∶1) and ethanolamine phosphate.

Metabolites of the polyamine pathway were not significantly altered over the nifurtimox time course, although decreased thiol levels (trypanothione disulphide and glutathionyl-cysteine disulphide) were observed, suggesting that oxidative stress may be induced on exposure to nifurtimox in agreement with previous studies [6], [7], [44], although the role of this stress in ultimate trypanocidal effect is uncertain. It is noted that this untargeted metabolomics approach is not suited for assessment of redox balance (as reduced thiols are oxidised during sample preparation and analysis), however and it is assumed that the observed disulphide levels are indicative of total thiol levels. The presence of oxidative stress may also explain the observed inhibition of glycolysis [45], and the decreased levels of arginine phosphate [46].

### NECT induced perturbations to the trypanosome's metabolome

We also investigated changes to the metabolome associated with exposure to eflornithine and nifurtimox simultaneously (Figure S4). The metabolome of NECT treated cells was measured using drug levels that were toxic in the monotherapies (500 µM for eflornithine and 60 µM for nifurtimox) and the time points used in the nifurtimox toxicity assay (0, 1, 2 and 5 hours), after which cells died without remaining viable for as long as studied in the eflornithine monotherapy study. The rapid reduction of Nifurtimox (within 1 hour) to the saturated open chain nitrile was still observed (Fig. 4B). This indicates that the nitroreductase activity known to be responsible for metabolic activation of nifurtimox [11] is not diminished in a short term response to eflornithine.

**Figure 5:**
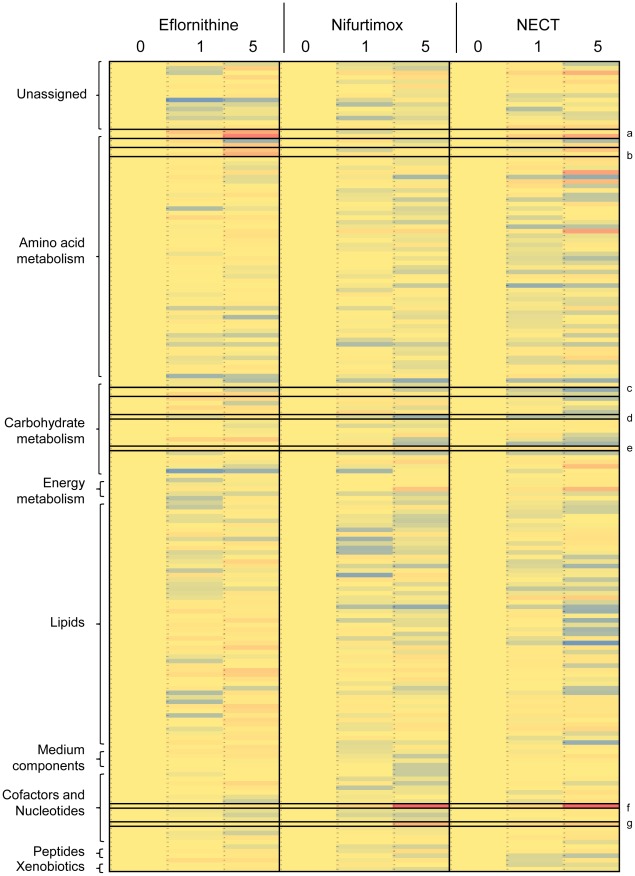
Heat map of metabolite levels in eflornithine, nifurtimox and NECT toxicity experiments. Blue represents a decrease in metabolite intensity, red an increase and yellow represents unchanged levels compared to the levels at the 0 hour time point. Metabolites are classified down the left hand side. Metabolites of interest are emphasised with black boxes - a: acetylornithine and ornithine, b: methylthioadenosine and cystathionine, c: succinate and malate, d: hexose 6-phosphate, e: pentose 5-phosphate, f: adenine and g: uracil.

The combination therapy showed qualitatively most of the same changes that were present in each of the monotherapies alone (Table 2 and Fig. 5). This indicates that both of the drugs are able to exert their individual effects and no additional effects were apparent using the combination. The eflornithine-induced changes to polyamine pathway metabolites were observed in the combination (Fig. 2), although the later effects of eflornithine could not be measured as cells died from nifurtimox toxicity before these were apparent. The nifurtimox-induced changes to nucleotides, glycolysis intermediates, deoxyribose and thiols were all observed to a similar extent in the combination treatment.

## Discussion

Understanding how small chemicals interfere with cellular metabolism is a critical part of modern drug development. Here we show how a relatively simple LC-MS based metabolomics platform can be used to identify drug modes of action in the causative agent of human African trypanosomiasis, *Trypanosoma brucei*. Using each of the drugs currently used in combination as a first line treatment against stage two HAT we reveal how modes of action of drugs can be rapidly ascertained.

At low levels of drug (sub IC_50_) specific changes to the metabolome can be detected as was evidenced with eflornithine. The data reveal very localised changes to the metabolome with little indication of broadly disseminated affects consistent with the theory that metabolic networks are generally robust to perturbations [47].

This study reveals the power of metabolomics for predicting the MOA of compounds with a metabolic (enzyme inhibition) mode of action. As ornithine accumulation and putrescine loss were the most significant changes between treated and untreated cells, ornithine decarboxylase emerges as the most likely target for this drug. In this case, the outcome was already known hence the follow up experiments e.g. showing that ODC is essential using gene knockout [48] and that addition of polyamines to the medium can bypass eflornithine toxicity [48] have already been performed. With unknown drugs, of course, these validation experiments are still required once the hypothesis has been set using metabolomics. The presence of the open chain trinitrile in nifurtimox-treated cells confirms the trypanosome-mediated metabolic activation of this drug, as was recently demonstrated following substantial targeted analysis of nifurtimox [11]. It will be of interest to extend studies to other current trypanocides and also to systematically include metabolomics in any test of action for compounds emerging from screens.

Eflornithine inhibited ODC relatively quickly with levels of ornithine and putrescine demonstrably altered after just five hours in toxic doses of drug. Trypanothione is a glutathione-spermidine adduct and its overall levels are diminished by around 73% prior to death in the eflornithine study, which is similar to the 66% reduction determined after eflornithine exposure *in vivo* [10], but it should be noted that many unrelated metabolites were also diminished at 48 hours. An advantage of the non-targeted metabolomics platform used here over a strictly targeted approach to report on individual metabolites is thus clear. Loss of putrescine and spermidine appears to contribute to cellular toxicity independently of their role in trypanothione biosynthesis as rescue experiments where spermidine is given exogenously to ODC knock down cells were unsuccessful [49]. Our studies indicate that eflornithine is trypanostatic for 48 hours, before killing the parasites after apparently compromising the membrane of the cell, as judged by a general loss of metabolites from the cell and particularly changes in lipid content. Since polyamines have been proposed to help stabilise membrane phospholipids [34], [35] this could indicate the actual cause of death following reduction in polyamine biosynthesis. *In vivo*, changes to membrane integrity would also expose new ligands to the immune systems, possibly explaining the need of an active immune system for optimal eflornithine activity [47]. Nifurtimox did not show the same depletion in membrane integrity prior to cell death.

The untargeted metabolomics approach was particularly useful for the identification of unexpected metabolites. Acetylated ornithine and putrescine have not been previously described in trypanosomes, and would likely not have been assessed with a classical targeted approach, but these results clearly show the presence of acetylated polyamines, and their dynamic relationships with polyamine levels, with N-acetyl ornithine correlating particularly well with ornithine. This metabolite has an unknown function within trypanosomes but appears to be created directly from ornithine transported into the cell. We have also shown that trypanosomes do not use classical arginase activity comparable to that found in related *Leishmania* spp. parasites to create ornithine from arginine but do have the ability to transport ornithine which is present in plasma and CSF, indicating that they probably fulfil ornithine needs by acquiring it directly from the host. Interestingly they can, nevertheless, convert arginine to ornithine, but apparently only when exogenous ornithine is not available.

An increase in sedoheptulose and sedoheptulose phosphate in eflornithine-treated cells was also of interest. Sedoheptulose phosphate is a seven carbon sugar of the pentose phosphate pathway, formed, along with glyceraldehyde 3-phosphate, from ribose 5-phosphate and xylulose 5-phosphate through transketolase (Tb927.8.6170) action. Transketolase activity, however, is absent in bloodstream-form trypanosomes [50], [51], although it is induced in parasites transforming between bloodstream and procyclic forms. It is possible, therefore, that the increase in sedoheptulose 7-phosphate could relate to transketolase being switched on in relation to the proposed induction of differentiation between slender bloodstream form and stumpy form organisms. Although the s427 strain used here does not differentiate to stumpy forms, other biochemical events such as the induction of enzymes usually repressed in the non-dividing stumpy stage may occur. Nifurtimox treatment did not induce any changes to sedoheptulose or its phosphate's levels.

Toxic doses of nifurtimox revealed alterations to levels of various nucleotide, carbohydrate and lipid metabolites. More work is required to ascertain why these metabolites' levels are altered with nifurtimox treatment and how these changes relate to death. However, our data reveal that this metabolomics approach can confirm previous findings that relate oxidative stress to nifurtimox treatment, and demonstrate the production of an open chain trinitrile metabolite in agreement with the proposed mechanism for the drug's selective activity against trypanosomes [11]. We show also that the appearance of this metabolite is relatively fast, being detectable within an hour of exposure.

The nifurtimox-eflornithine combination therapy, which was previously assumed to be synergistic, was shown to be mildly antagonistic *in vitro*. The theory behind synergy was based on the assumption that eflornithine would decease cellular trypanothione levels thus decreasing the ability of these cells to defend against oxidative stress. Since nifurtimox was generally believed to exert an effect through generation of reactive oxygen species [6], [7] it followed that eflornithine treated cells would show enhanced susceptibility to nifurtimox. However, the metabolic perturbations observed in this study suggest that oxidative stress is not the primary MOA for either drug (despite some indication of oxidative stress observed with nifurtimox), and if nifurtimox actually acts through production of the reactive open chain trinitrile and its ability to covalently modify macromolecules, then the proposed synergy would not exist. It should be noted too, however, that our studies *in vitro* need not reflect the situation *in vivo* where pharmacokinetic factors lead to very different exposure of parasites to drug and where other host related factors, not least the immune response, contribute to effects of the drugs, although in mice at least neither drug facilitates entry of the other into the brain.

A potential reason why the drug combination is mildly antagonistic *in vitro* could relate to the activation of nifurtimox and its target based effects depending upon growth status of the cell. There was no evidence that activation of nifurtimox was reduced in the eflornithine co-treated cells. Instead, therefore, it is possible that cells entering a state of reduced growth are less affected by the impact of nifurtimox on energy and nucleic acid metabolism. This hypothesis was supported by the antagonism to nifurtimox seen with the trypanostatic purine analogues NA42 and NA134 [36].

The examples we provide here demonstrate how a relatively simple metabolomics platform can elucidate the mode of action of a drug in a relatively short time frame. This study shows that our metabolomics platform yields hypothesis-free data that confirm the known MOA of eflornithine and create testable hypotheses for the nifurtimox MOA as well as confirming a lack of synergy of NECT. The approach we provide here can be readily adapted for other drugs and cellular systems.

## Supporting Information

Figure S1 **Ideom File showing all metabolites identified in low dose eflornithine treatment experiment:** Metabolites are listed and the file in an Excel format can be read according to instructions at reference 52.
(XLSB)

Figure S2 **Ideom File showing all metabolites identified in high dose eflornithine treatment experiment.** Metabolites are listed and the file in an Excel format can be read according to instructions at reference 52.
(XLSB)

Figure S3 **Ideom File showing all metabolites identified in nifurtimox treatment experiment:** Metabolites are listed and the file in an Excel format can be read according to instructions at reference 52.
(XLSB)

Figure S4 **Ideom File showing all metabolites identified in NECT treatment experiment.** Metabolites are listed and the file in an Excel format can be read according to instructions at reference 52.
(XLSB)

## References

[1] Brun R, Blum J, Chappuis F, Burri C (2010). Human African trypanosomiasis.. Lancet.

[2] Barrett MP, Boykin DW, Brun R, Tidwell RR (2007). Human African trypanosomiasis: pharmacological re-engagement with a neglected disease.. Br J Pharmacol.

[3] Grishin NV, Osterman AL, Brooks HB, Phillips MA, Goldsmith EJ (1999). X-ray structure of ornithine decarboxylase from *Trypanosoma brucei*: the native structure and the structure in complex with alpha-difluoromethylornithine.. Biochemistry.

[4] Poulin R, Lu L, Ackermann B, Bey P, Pegg AE (1992). Mechanism of the Irreversible Inactivation of Mouse Ornithine Decarboxylase by Alpha-Difluoromethylornithine - Characterization of Sequences at the Inhibitor and Coenzyme Binding-Sites.. J Biol Chem.

[5] Priotto G, Kasparian S, Mutombo W, Ngouama D, Ghorashian S (2009). Nifurtimox-eflornithine combination therapy for second-stage African *Trypanosoma brucei gambiense* trypanosomiasis: a multicentre, randomised, phase III, non-inferiority trial.. Lancet.

[6] Docampo R, Stoppani AO (1979). Generation of superoxide anion and hydrogen peroxide induced by nifurtimox in *Trypanosoma cruzi*.. Arch Biochem Biophys.

[7] Boiani M, Piacenza L, Hernandez P, Boiani L, Cerecetto H (2010). Mode of action of Nifurtimox and N-oxide-containing heterocycles against *Trypanosoma cruzi*: Is oxidative stress involved?. Biochem Pharmacol.

[8] Fairlamb AH, Blackburn P, Ulrich P, Chait BT, Cerami A (1985). Trypanothione: a novel bis(glutathionyl)spermidine cofactor for glutathione reductase in trypanosomatids.. Science.

[9] Bacchi CJ, Garofalo J, Mockenhaupt D, McCann PP, Diekema KA (1983). *In vivo* effects of alpha-DL-difluoromethylornithine on the metabolism and morphology of *Trypanosoma brucei brucei*.. Mol Biochem Parasitol.

[10] Fairlamb AH, Henderson GB, Bacchi CJ, Cerami A (1987). *In vivo* effects of difluoromethylornithine on trypanothione and polyamine levels in bloodstream forms of *Trypanosoma brucei*.. Mol Biochem Parasitol.

[11] Hall BS, Bot C, Wilkinson SR (2011). Nifurtimox activation by trypanosomal type I nitroreductases generates cytotoxic nitrile metabolites.. J Biol Chem.

[12] Jeganathan S, Sanderson L, Dogruel M, Rodgers J, Croft S (2011). The distribution of nifurtimox across the healthy and trypanosome-infected murine blood-brain and blood-cerebrospinal fluid barriers.. J Pharmacol Exp Ther.

[13] Vincent IM, Creek D, Watson DG, Kamleh MA, Woods DJ (2010). A molecular mechanism for eflornithine resistance in African trypanosomes.. PLoS Pathog.

[14] Hughes B (2010). 2009 FDA drug approvals.. Nat Rev Drug Discov.

[15] Creek D, Anderson J, McConville MJ, Barrett MP (2011). Metabolomic analysis of trypanosomatid protozoa.. Mol Biochem Parasitol Epub ahead of print.

[16] Barrett MP, Bakker BM, Breitling R (2010). Metabolomic systems biology of trypanosomes.. Parasitology.

[17] Creek DJ, Jankevics A, Breitling R, Watson DG, Barrett MP, Burgess KE (2011). Towards Global Metabolomics Analysis with Liquid Chromatography-Mass Spectrometry: Improved Metabolite Identification by Retention Time Prediction.. Anal Chem Epub ahead of print.

[18] Kamleh A, Barrett MP, Wildridge D, Burchmore RJ, Scheltema RA (2008). Metabolomic profiling using Orbitrap Fourier transform mass spectrometry with hydrophilic interaction chromatography: a method with wide applicability to analysis of biomolecules.. Rapid Commun Mass Spectrom.

[19] Burgess KE, Creek D, Dewsbury P, Cook K, Barrett MP (2011). Semi-targeted analysis of metabolites using capillary-flow ion chromatography coupled to high-resolution mass spectrometry.. Rapid Commun Mass Spectrom.

[20] Allen J, Davey HM, Broadhurst D, Rowland JJ, Oliver SG (2004). Discrimination of modes of action of antifungal substances by use of metabolic footprinting.. Appl Environ Microbiol.

[21] Aranibar N, Singh BK, Stockton GW, Ott KH (2001). Automated mode-of-action detection by metabolic profiling.. Biochem Biophys Res Commun.

[22] Bando K, Kunimatsu T, Sakai J, Kimura J, Funabashi H (2010). GC-MS-based metabolomics reveals mechanism of action for hydrazine induced hepatotoxicity in rats.. J Appl Toxicol.

[23] Sun L, Li HM, Seufferheld MJ, Walters KR, Margam VM (2011). Systems-scale analysis reveals pathways involved in cellular response to methamphetamine.. PLoS One.

[24] van Brummelen AC, Olszewski KL, Wilinski D, Llinás M, Louw AI (2009). Co-inhibition of *Plasmodium falciparum* S-adenosylmethionine decarboxylase/ornithine decarboxylase reveals perturbation-specific compensatory mechanisms by transcriptome, proteome, and metabolome analyses.. J Biol Chem.

[25] Hirumi H, Doyle JJ, Hirumi K (1977). Cultivation of Blood-Stream *Trypanosoma-Brucei*.. Bulletin of the World Health Organization.

[26] Raz B, Iten M, Grether-Buhler Y, Kaminsky R, Brun R (1997). The Alamar Blue assay to determine drug sensitivity of African trypanosomes (*T.b. rhodesiense* and *T.b. gambiense*) in vitro.. Acta Tropica.

[27] Carter NS, Fairlamb AH (1993). Arsenical resistant trypanosomes lack an unusual adenosine transporter.. Nature.

[28] Saunders EC, DE Souza DP, Naderer T, Sernee MF, Ralton JE (2010). Central carbon metabolism of Leishmania parasites.. Parasitology.

[29] t'Kindt R, Jankevics A, Scheltema RA, Zheng L, Watson DG (2010). Towards an unbiased metabolic profiling of protozoan parasites: optimisation of a Leishmania sampling protocol for HILIC-orbitrap analysis.. Anal Bioanal Chem.

[30] Scheltema RA, Jankevics A, Jansen RC, Swertz MA, Breitling R (2011). PeakML/mzMatch: a file format, Java library, R library, and tool-chain for mass spectrometry data analysis.. Anal Chem.

[31] Smith CA, Want EJ, O'Maille G, Abagyan R, Siuzdak G (2006). XCMS: processing mass spectrometry data for metabolite profiling using nonlinear peak alignment, matching, and identification.. Anal Chem.

[32] Rodenko B, van der Burg AM, Wanner MJ, Kaiser M, Brun R (2007). 2,N6-disubstituted adenosine analogs with antitrypanosomal and antimalarial activities.. Antimicrob Agents Chemother.

[33] Lanteri CA, Trumpower BL, Tidwell RR, Meshnick SR (2004). DB75, a novel trypanocidal agent, disrupts mitochondrial function in *Saccharomyces cerevisiae*.. Antimicrob Agents Chemother.

[34] Serricchio M, Butikofer P (2011). *Trypanosoma brucei*: a model micro-organism to study eukaryotic phospholipid biosynthesis.. FEBS J.

[35] Hernandez SM, Sanchez MS, de Tarlovsky MN (2006). Polyamines as a defense mechanism against lipoperoxidation in *Trypanosoma cruzi*.. Acta Trop.

[36] Souzu H (1986). Fluorescence Polarization Studies on *Escherichia-Coli* Membrane Stability and Its Relation to the Resistance of the Cell to Freeze-Thawing.2. Stabilization of the Membranes by Polyamines.. Biochimt Biophys Acta.

[37] Hasne MP, Barrett MP (2000). Transport of methionine in *Trypanosoma brucei brucei*.. Mol Biochem Parasitol.

[38] da Silva ER, Castilho TM, Pioker FC, Tomich de Paula Silva CH, Floeter-Winter LM (2002). Genomic organisation and transcription characterisation of the gene encoding *Leishmania (Leishmania) amazonensis* arginase and its protein structure prediction.. Int J Parasitol.

[39] Riley E, Roberts SC, Ullman B (2011). Inhibition profile of *Leishmania mexicana* arginase reveals differences with human arginase I.. Int J Parasitol.

[40] Gaur U, Roberts SC, Dalvi RP, Corraliza I, Ullman B (2007). An effect of parasite-encoded arginase on the outcome of murine cutaneous leishmaniasis.. J Immunol.

[41] da Silva ER, da Silva MF, Fischer H, Mortara RA, Mayer MG (2008). Biochemical and biophysical properties of a highly active recombinant arginase from *Leishmania (Leishmania) amazonensis* and subcellular localization of native enzyme.. Mol Biochem Parasitol.

[42] Albrecht AM, Vogel HJ (1964). Acetylornithine delta-transaminase. Partial purification and repression behavior.. J Biol Chem.

[43] Xu Y, Glansdorff N, Labedan B (2006). Bioinformatic analysis of an unusual gene-enzyme relationship in the arginine biosynthetic pathway among marine gamma proteobacteria: implications concerning the formation of N-acetylated intermediates in prokaryotes.. BMC Genomics.

[44] Enanga B, Ariyanayagam MR, Stewart ML, Barrett MP (2003). Activity of megazol, a trypanocidal nitroimidazole, is associated with DNA damage.. Antimicrob Agents Chemother.

[45] Penketh PG, Klein RA (1986). Hydrogen peroxide metabolism in *Trypanosoma brucei*.. Mol Biochem Parasitol.

[46] Miranda MR, Canepa GE, Bouvier LA, Pereira CA (2006). *Trypanosoma cruzi*: Oxidative stress induces arginine kinase expression.. Exp Parasitol.

[47] Bitonti AJ, McCann PP, Sjoerdsma A (1986). Necessity of antibody response in the treatment of African trypanosomiasis with alpha-difluoromethylornithine.. Biochem Pharmacol.

[48] Li F, Hua SB, Wang CC, Gottesdiener KM (1998). *Trypanosoma brucei brucei*: characterization of an ODC null bloodstream form mutant and the action of alpha-difluoromethylornithine.. Exp Parasitol.

[49] Xiao Y, McCloskey DE, Phillips MA (2009). RNA interference-mediated silencing of ornithine decarboxylase and spermidine synthase genes in *Trypanosoma brucei* provides insight into regulation of polyamine biosynthesis.. Eukaryotic Cell.

[50] Cronín CN, Nolan DP, Voorheis HP (1989). The enzymes of the classical pentose phosphate pathway display differential activities in procyclic and bloodstream forms of *Trypanosoma brucei*.. FEBS Lett.

[51] Stoffel SA, Alibu VP, Hubert J, Ebikeme C, Portais JC (2011). Transketolase in *Trypanosoma brucei*.. Mol Biochem Parasitol.

[52] Yun O, Priotto G, Tong J, Flevaud L, Chappuis F (2010). NECT is next: implementing the new drug combination therapy for *Trypanosoma brucei gambiense* sleeping sickness.. PLoS Negl Trop Dis.

[53] Sands M, Kron MA, Brown RB (1985). Pentamidine: a review.. Rev Infect Dis.

[54] Voogd TE, Vansterkenburg EL, Wilting J, Janssen LH (1993). Recent research on the biological activity of suramin.. Pharmacol Rev.

